# Colovesical Fistula as a Late Complication of Ingestion of a Foreign Body

**DOI:** 10.5334/jbsr.3426

**Published:** 2024-02-06

**Authors:** Bénédicte Verbrugge, Adelard De Backer, Koenraad Nieboer

**Affiliations:** 1 Vrije Universiteit Brussel (VUB), Universitair Ziekenhuis Brussel (UZ Brussel), Department of Radiology, Laarbeeklaan 101, 1090 Brussels, Belgium, Email: benedicte.verbrugge@gmail.com; 2 Vrije Universiteit Brussel (VUB), Universitair Ziekenhuis Brussel (UZ Brussel), Department of Radiology, Laarbeeklaan 101, 1090 Brussels, Belgium, Email: Adelard.DeBacker@uzbrussel.be; 3 Vrije Universiteit Brussel (VUB), Universitair Ziekenhuis Brussel (UZ Brussel), Department of Radiology, Laarbeeklaan 101, 1090 Brussels, Belgium, Email: Koenraad.Nieboer@uzbrussel.be

**Keywords:** Colovesical Fistula, Foreign Body, CT

## Abstract

*Teaching point:* Ingestion of a foreign body is a rare cause of a colovesical fistula.

## Case History

**Figure 1:**
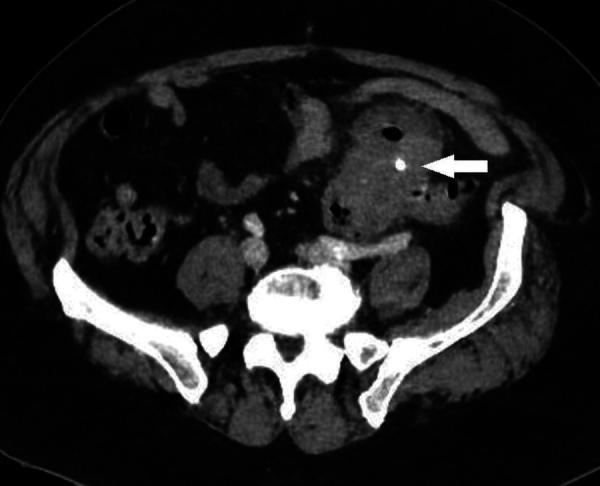

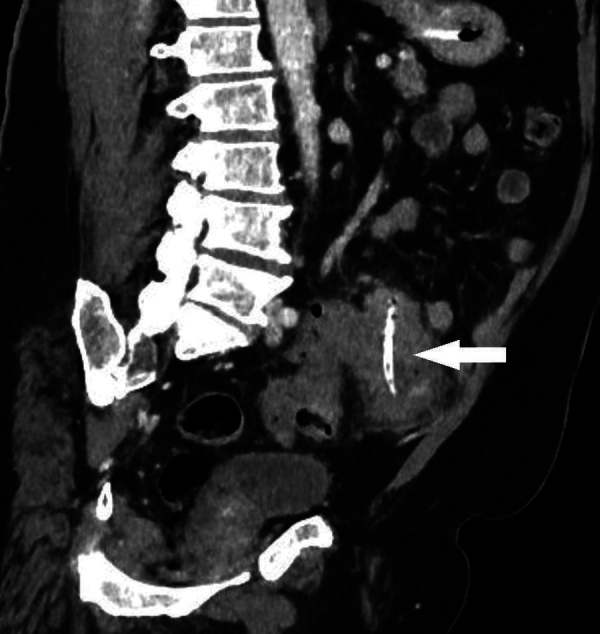
Axial and sagittal image demonstrating foreign body perforating sigmoid colon.

A 64-year-old male patient presented with a progressive onset of abdominal pain. Two months earlier, the patient had an accidental ingestion of a chicken bone. Clinical examination showed most pain was located in the left fossa without rebound. Lab values showed increased C-reactive protein and leukocytosis. Abdominal computed tomography (CT) demonstrated a foreign body with bone density (Figure 1, arrow) perforating the sigmoid colon and associated inflammatory thickening of the sigmoid with adjacent inflammatory mass and abscess. The bone was removed endoscopically.

**Figure 2:**
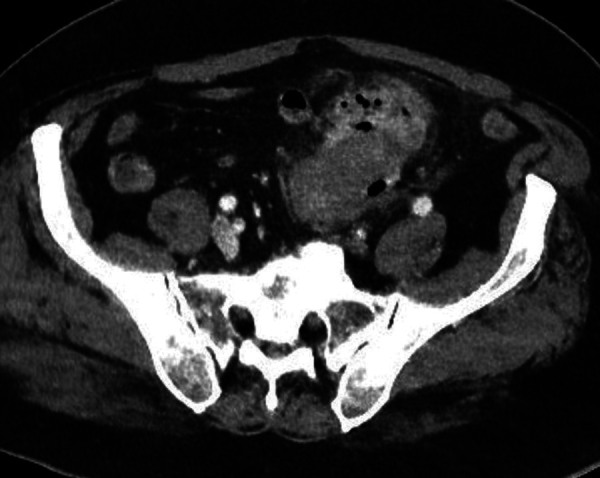

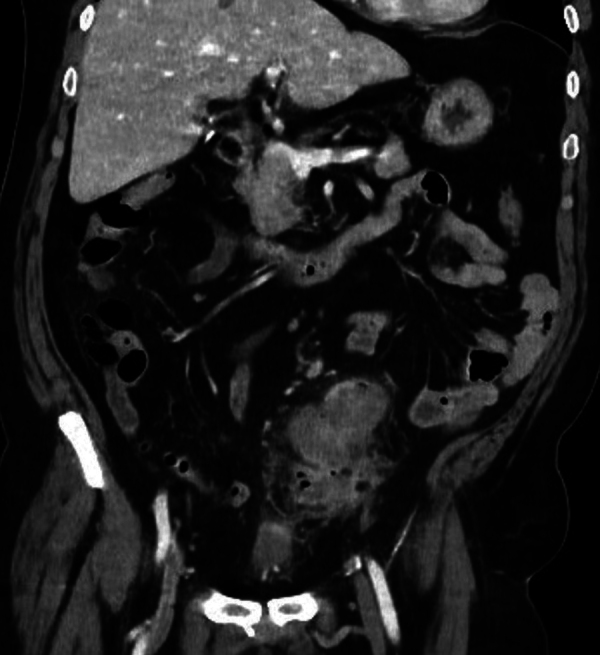
Axial and coronal image showing inflammatory mass adjacent to sigmoid.

**Figure 3:**
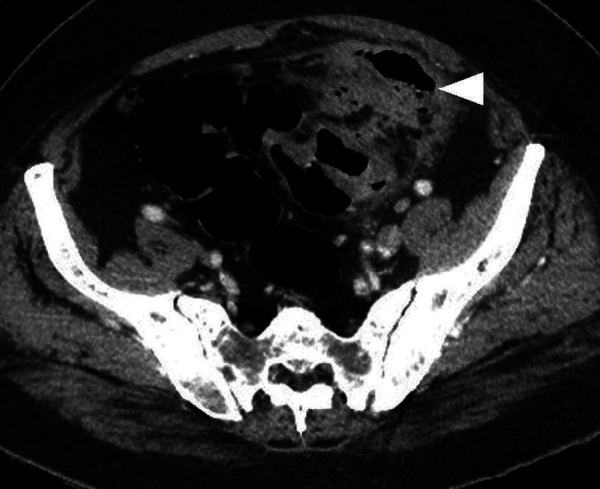

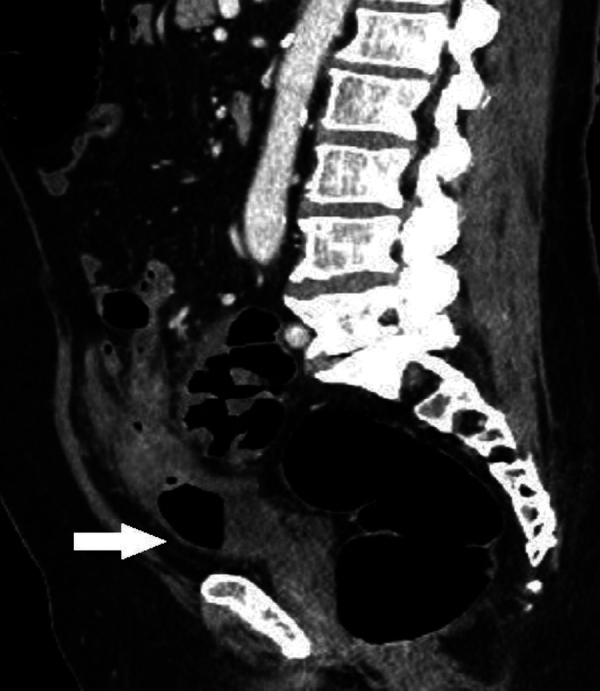
Inflammatory mass and air in bladder resulting from colovesical fistula.

Despite conservative treatment with antibiotics, the patient was seen 2 weeks later with persisting abdominal pain. CT showed a persisting inflammatory mass adjacent to the sigmoid (Figure 2). The patient refused surgical intervention, and conservative treatment was continued. One month later, patient presented with urosepsis. CT was repeated and showed an extensive inflammatory mass adjacent to the sigmoid with intralesional air bubbles in continuity with a thickened bladder wall (Figure 3, arrowhead). The presence of air was noted in the bladder (Figure 3, arrow). The diagnosis of a colovesical fistula (CVF) as a late complication of iatrogenic bowel wall perforation with an abscess was made.

## Comments

A CVF usually occurs as the result of an inflammatory process between the colon and the bladder complicated by abscess formation. This rare condition is most often seen in cases of sigmoid diverticulitis, which is complicated by abscess formation extending to the bladder wall. Less frequently causes are neoplasia of the colon and bladder, previous surgery (e.g., radical prostatectomy), or pelvic radiation therapy. The presence of a CVF after accidental ingestion of a foreign body resulting in perforation of the bowel wall and abscess formation is rarely seen [1].

CT is the examination of choice for the detection of perforating foreign bodies. A thickened bowel wall, adjacent fatty stranding, an inflammatory mass with or without an abscess, localized pneumoperitoneum, or an associated intestinal obstruction are indirect signs. The final diagnosis is made by direct visualization of the foreign body perforating the bowel wall. In our patient, diverticulosis of the sigmoid colon was present. Chicken bone with the tip fixed into a diverticulum and bowel peristalsis probably caused perforation with subsequent inflammation.

CT is the imaging modality of choice to diagnose CVF. Pericolic inflammation in continuity with the bladder associated with air in the bladder is a pathognomonic sign. Peroral contrast intake or rectal contrast administration with leakage from the bowel into the bladder is also a key finding in diagnosis. In the past, fluoroscopic radiography, such as water-soluble contrast enema, cystography, or intravenous urography was also used.

Surgery is the treatment option of choice for CVF. Fistulae due to inflammation of the sigmoid are generally treated with resection of the inflamed segment. Repair of the bladder is only performed when large visible defects are present. Conservative management is less indicated because of its high morbidity and mortality.
